# A microfluidic approach for synchronous and nondestructive study of the permeability of multiple oocytes

**DOI:** 10.1038/s41378-020-0160-4

**Published:** 2020-07-27

**Authors:** Zhongrong Chen, Kashan Memon, Yunxia Cao, Gang Zhao

**Affiliations:** 1 Department of Electronic Science and Technology, University of Science and Technology of China, Hefei, 230027 China; 2 Reproductive Medicine Center, Department of Obstetrics and Gynecology, The First Affiliated Hospital of Anhui Medical University, Hefei, 230022 China; 3 Anhui Province Key Laboratory of Reproductive Health and Genetics, Anhui Provincial Engineering Technology Research Center for Biopreservation and Artificial Organs, Anhui Medical University, Hefei, 230022 China

**Keywords:** Nanoscale devices, Engineering

## Abstract

Investigation of oocyte membrane permeability plays a crucial role in fertility preservation, reproductive medicine, and reproductive pharmacology. However, the commonly used methods have disadvantages such as high time consumption, low efficiency, and cumbersome data processing. In addition, the developmental potential of oocytes after measurement has not been fully validated in previous studies. Moreover, oocytes can only maintain their best status in vitro within a very limited time. To address these limitations, we developed a novel multichannel microfluidic chip with newly designed micropillars that provide feasible and repeatable oocyte capture. The osmotic responses of three oocytes at different or the same cryoprotectant (CPA) concentrations were measured simultaneously, which greatly improved the measurement efficiency. Importantly, the CPA concentration dependence of mouse oocyte membrane permeability was found. Moreover, a neural network algorithm was employed to improve the efficiency and accuracy of data processing. Furthermore, analysis of fertilization and embryo transfer after perfusion indicated that the microfluidic approach does not damage the developmental potential of oocytes. In brief, we report a new method based on a multichannel microfluidic chip that enables synchronous and nondestructive measurement of the permeability of multiple oocytes.

## Microfluidics: Measuring oocyte permeability

A multichannel microfluidic device enables nondestructive measurement of oocyte permeability. Knowing the permeability of oocyte membranes is particularly important in the context of the use of cryoprotectants for the storage of oocytes for human fertility. However, oocytes are particularly sensitive to osmotic pressure generated by the freezing that accompanies the use of cryoprotectants. Understanding membrane permeability is therefore important in controlling the freezing process. Here, a team led by Gang Zhao from University of Science and Technology of China report a microfluidic device that can simultaneously determine the permeability of multiple oocytes, ensuring accurate measurement. The key design is the use of micropillars, which capture the oocytes. They also demonstrate that oocytes are not damaged and their development is not impaired.

## Introduction

Oocyte permeability refers to the water conductivity (*L*_p_) and other small molecule permeability (*P*_s_) of the oocyte membrane^1^. Investigation of oocyte membrane permeability plays a crucial role in fertility preservation, reproductive medicine, and reproductive pharmacology^2,3^. These studies significantly promote the development of assisted reproductive technology (ART)^4^. In particular, oocyte cryopreservation^5–7^ has attracted ever-increasing interest^8^ and has been considered one of the most promising techniques to preserve human fertility^9^. However, despite encouraging achievements in successful fertilization and birth, the pregnancy success rate of cryopreserved oocytes remains quite low^10,11^, which is because current methods for cryopreservation still have shortcomings^12^. Generally, the oocyte cryopreservation process includes cryoprotectant (CPA) addition/removal and a freeze-thaw cycle^1,8^. Oocytes are more sensitive to extracellular osmotic pressure variations than other cells^13^. During cryopreservation, oocytes are more susceptible to osmotic shock to CPA solutions, as well as extra/intracellular ice formation-induced mechanical injury^1,14^. Therefore, accurate measurement of oocyte membrane permeability is of great significance in the field of oocyte cryopreservation and ART^12^.

The permeability of the oocyte membrane has previously been extensively investigated^12,15–26^. Traditionally, micropipette perfusion and direct microscopic observation were utilized to quantify the permeability parameters of the oocyte membrane^20,27,28^. With the development of microfluidics^29–34^, microfluidic devices have shown great advantages and prospects in cell research^35–38^. Diverse microfluidic devices have been developed to measure the permeability of oocytes instead of using manual methods. For example, polydimethylsiloxane (PDMS) microfluidic platforms were reported to quantitatively measure oocyte volume response during CPA addition^16,18^. Recently, a microfluidic platform with precise temperature control was reported for studying the temperature dependence of oocyte permeability^39^. In addition, a non-PDMS-based microscope diffusion chamber and microperfusion chamber were reported to measure the permeability of oocytes^27,40^. Zhao’s group recently developed a novel sandwich-structured microfluidic perfusion approach for accurately characterizing the permeability of human oocytes and achieving high-quality oocyte selection^12,41^. However, all of the above methods can only measure a single oocyte or sole CPA concentration at one time. These methods are labor intensive or have low efficiency, which greatly restricts their applications^12^. In addition, the developmental potential of measured oocytes has not been fully validated in the above studies. As is known, oocytes are a precious cell type and can only maintain normal viability within several hours after extraction^42,43^. The developmental potential of oocytes also has a profound impact on the early development and pregnancy of embryos^41^. Therefore, there is an urgent need for a device capable of simultaneously and nondestructively measuring the membrane permeability of multiple oocytes. In addition, to the best of our knowledge, the effect of CPA concentration on the permeability of oocyte membranes has received less attention and has not been fully investigated^44^.

Herein, we present a new microfluidic approach that is suitable for the simultaneous study of the permeability of multiple oocytes. The newly designed micropillars in the microchannel support oocyte automatic capture while avoiding undesired influences on extracellular CPA mixing. The chip provides simultaneous measurement of three oocytes exposed to three independent osmotic shifts, thereby making it possible to investigate the CPA concentration-dependent of oocyte membrane permeability. In addition, the chip can simultaneously measure three oocytes exposed to the same osmotic shift, which greatly improves the measurement efficiency. Moreover, a neural network algorithm is employed to improve the efficiency and accuracy of data processing. Furthermore, subsequent fertilization and embryo transfer (in vitro fertilization and embryo transfer (IVF-ET)) indicate that the microfluidic approach does not impair the developmental potential of oocytes. These special features can enable nondestructive and high-efficiency measurement of oocyte permeability and CPA concentration dependence. We are confident that this novel microfluidic approach has great application prospects in the field of fertility preservation, reproductive medicine and reproductive pharmacology.

## Results

### Microfluidic chip and its system

**Fig. 1:**
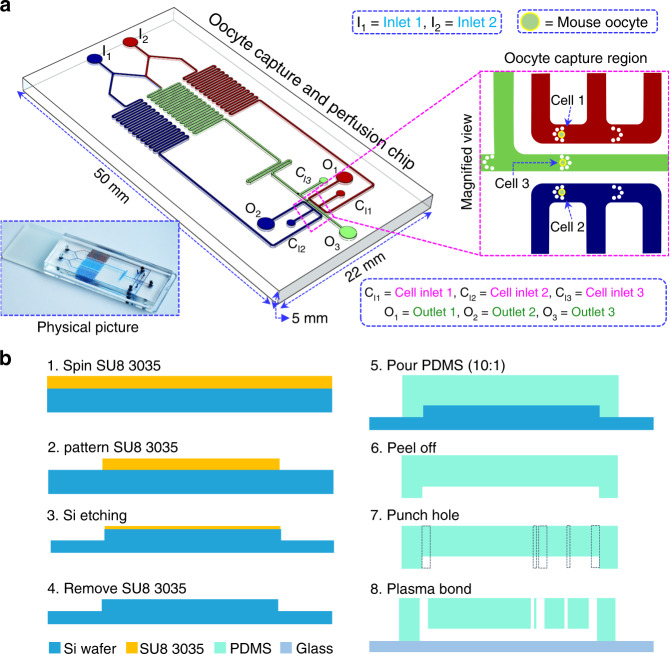
Schematic illustration of the microfluidic chip. **a** Schematic diagram of the microfluidic perfusion chip and experimental principle. **b** The main steps of microfluidic chip fabrication.

The microfluidic chip is shown in Fig. 1a. The channel sizes are 200 and 150 μm (*W* × *H*). Two inlet (I_1_, I_2_) paths are designed with a symmetrical structure, and the distance from the inlet to the outlet is the same to ensure the same resistance. A serpentine channel is designed to allow the solution to mix thoroughly and achieve different concentrations, which can be used for investigating permeability concentration dependence. The structure for oocyte capture, each channel has two micropillar obstacles. The size of each micropillar is 20 μm in radius and 150 μm in height. The obstacles set here are to capture oocytes, to prevent the oocytes from entering the serpentine channel, and to retrieve them easily. Moreover, the newly designed micropillars can avoid undesired influences on extracellular CPA mixing. During the experiment, oocytes are injected and retrieved from cell inlets (C_I1_, C_I2_, and C_I3_). Figure 1b shows the main procedure of chip fabrication.

As shown in Fig. S1, the whole system mainly includes a BDS series inverted biological microscope (Aote Optics, Chongqing, China), microfluidic chip, and high-precision programmable syringe pumps (WK-101P, Nanjing Anerke Electronic Technology, Nanjing, China). The microscope is equipped with an industrial camera (KP-2307 HD, KOPPACE, Shenzhen, China) and connected to computer software to observe and record the morphological changes of the oocytes. In this system, silicone hoses are used to connect the syringe pumps to the inlets of the microfluidic chip. These silicone hoses are of a certain thickness, which can improve the anti-interference ability and reduce the disturbance. The oocyte inlet channel is connected to the cell transplantation hose via a 0.7 mm needle.

### Simulation of the CPA solution concentration profile and flow in the microfluidic channel

**Fig. 2:**
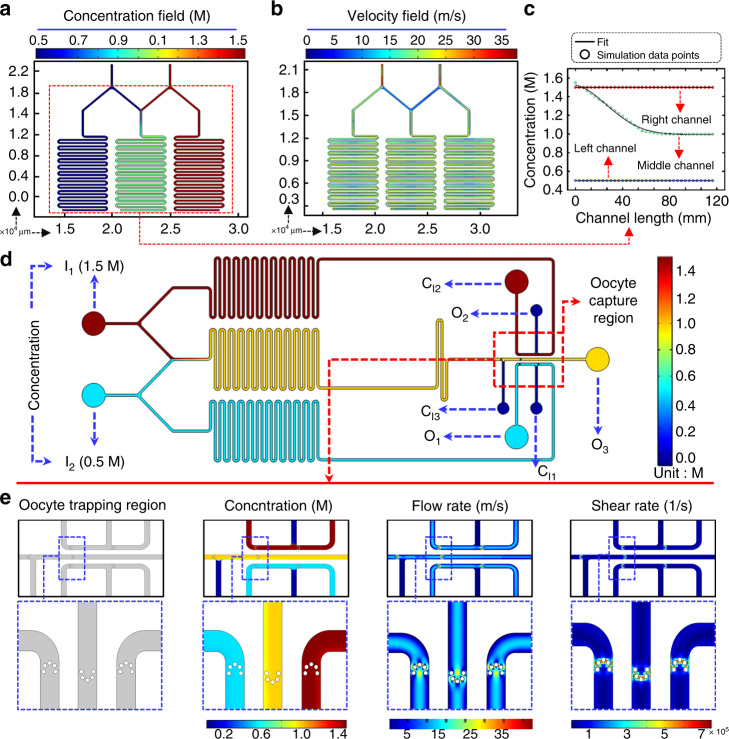
Simulation results of the CPA solution concentration profile and flow in the microfluidic channel. **a** Concentration gradient distribution of CPA in the serpentine channel. **b** Velocity profile of CPA inside the serpentine channel. **c** Simulated concentration profile along the serpentine channel. **d** Concentration profile in the whole microfluidic chip. **e** Simulation of the oocyte capture region, including the concentration profile, flow rate, and shear rate.

For the simulation results, the geometrical parameters and their boundary conditions are similar to those of the experimental microfluidic chip, as shown in Fig. S2. To ensure the accuracy and efficiency of the experimental model, finite element method (FEM) was used to obtain the variation in solution concentration at several specific points (P1 to P31). The simulated concentration changes along the mixing channel at these specific points are shown in Table S1. The concentrations at inlet1 and inlet2 are 1.5 and 0.5 M, respectively. Both inlets are set to the same flow rate of 10 μL/min. As shown in Fig. 2a, two solutions gradually mix when flowing through the intermediate channel, and the characteristics of the laminar flow in the microchannel appear at the initial intersection. When driven along the respective paths, due to the concentration difference, the solute diffuses at the boundary of the contact. After a certain length of the serpentine channel, the solution gradually mixes and reaches equilibrium. The velocity profiles of the solution flow in the three serpentine channels are almost the same (Fig. 2b). The final concentration of the middle channel is the average of the initial concentrations at the two inlets, as shown in Fig. 2c. These results demonstrate that the concentration gradient generation structure of the chip can effectively achieve different concentrations, and when reaching the oocyte capture region, the solution has reached the required consistency. Figure 2d shows the CPA solution concentration profile in the whole microfluidic chip. Since the size of the microchannel is not very large relative to mouse oocytes, the replacement of the solution around the oocytes can be seen as a uniform process. To further investigate the rationality of the oocyte capture region design, the detailed simulation results are shown in Fig. 2e. This subfigure displays the detailed concentration profile, flow rate and shear rate of the oocyte capture region. It should be noted that optimization design of the oocyte capture region was conducted. Three other different capture structures were designed and modeled to compare the pros and cons, as shown in Fig. S3. The results demonstrate that the design that we used is much better than other designs because the flow rate and shear rate are both optimal and have minimal impact on oocytes.

### Characterization of the solution concentration change in the microfluidic channel

**Fig. 3:**
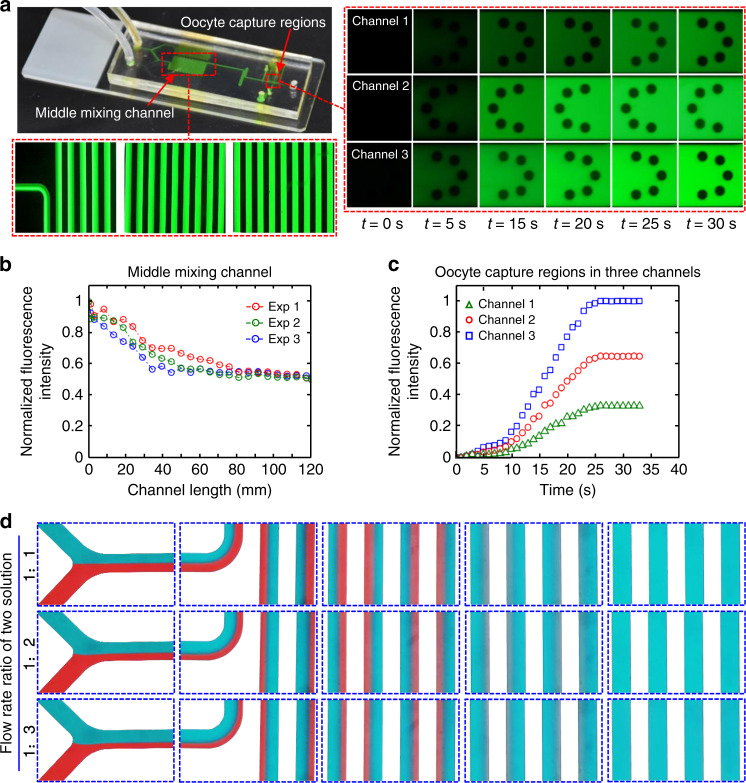
Characterization of solution concentration change in microfluidic channels. **a** Fluorescence map corresponding to the mixing channel and fluorescence map corresponding to three oocyte capture regions with different concentrations. **b** Change in fluorescence intensity along the mixing channel. **c** Change in fluorescence intensity at three oocyte capture regions. **d** Two dye solutions mixed in the mixing channel at different flow rate ratios.

To verify the validity of the FEM, the solution flow in the microfluidic channel was verified by fluorescent dye and food dye solutions. Two fluorescent dye solutions were prepared by adding two different doses of sodium fluorescein to an aqueous solution. These solutions exhibit green fluorescence under blue excitation light. Two syringe pumps were simultaneously turned on to inject two concentrations of fluorescent dye solution into the microchannel, and the mixing of the solution was judged by the fluorescence intensity at different positions. The fluorescence images at different locations of the channel were obtained by software photographing, and then, acquired images were further processed to obtain the fluorescence intensity graph, as shown in Fig. 3a. This subfigure shows the fluorescent solution flowing through the microchannels, and the two partially enlarged views represent the middle mixing channel and the oocyte capture locations. Under a fluorescence microscope (BX53, Olympus, Tokyo, Japan), the process of gradual mixing between two solutions can be seen in the microchannel. Three oocyte capture locations also showed different fluorescence intensity variations. To further obtain the quantitative results of the fluorescence intensity, different positions were selected along the mixing channel to measure the fluorescence intensity variation. The normalization fluorescence intensity curve (Fig. 3b) indicates that the fluorescence intensity at the exit of the serpentine mixing channel decreases to half of the initial maximum intensity, which means that the mixing of the two solutions has been completed, and the mixed solution with the required concentration will reach the oocyte capture region. Meanwhile, the normalized fluorescence intensity change at three oocyte capture locations (Fig. 3c) indicates that the concentration of the three capture locations has multiple relationships, which is consistent with our design philosophy. Moreover, the fluorescence map corresponding to three oocyte capture locations with the same concentration and fluorescence intensity variation at three oocyte capture locations is shown in Fig. S4.

In addition, a two-color food dye solution mixing experiment in the middle channel was conducted. As shown in Fig. 3d, two kinds of food dye are utilized to prepare solutions, and three flow rate ratios are selected. The two solutions occupy different spaces of the channel at first and then mix along the serpentine channel. Finally, the solution becomes one color. With different ratios, the final color tone is different. These results also demonstrate the practicability and rationality of the microfluidic chip.

### Neural network method for image processing

**Fig. 4:**
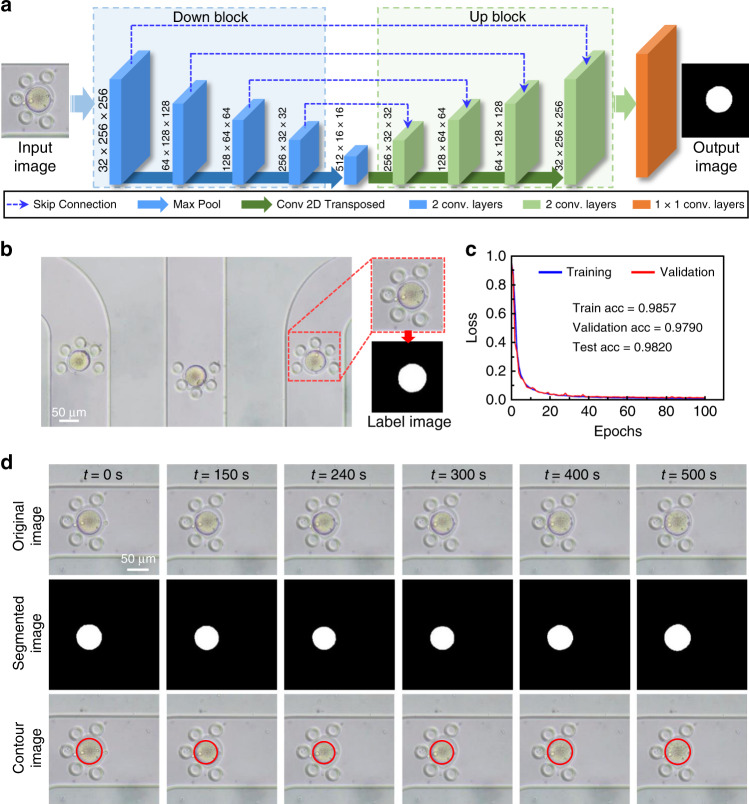
Neural network architecture and image processing performance. **a** Neural network architecture for cell image segmentation. **b** Typical image frame of three oocytes captured in the microfluidic chip. A 256 × 256 pixel image with an oocyte was processed as the data set of the network, and the label image corresponded to the data set. **c** The change in the loss function of the training set and validation set with epochs. **d** Typical photomicrographs of oocyte volume response to CPA, segmented images obtained from neural network and contour images drawn according to segmented results.

The neural network architecture for oocyte image segmentation consists of two main parts: the contracting part (Down Block) in the blue dotted box and the expanding part (Up Block) in the green dotted box (Fig. 4a). Blue dotted arrow lines indicate the skip connections. The blue and green bars both include two convolutional layers, while orange only includes one. The Up Block model supports the construction of accurate results, which is similar to the Down Block. With skip connections, information can be directly transferred from the Down Block to the Up Block.

The most important part of the neural network method is to train the network to achieve precise results^39^. The data used for training are prepared as shown in Fig. 4b. An image containing one oocyte (256 × 256 pixel) is taken out as the data set, and the corresponding label image represents a correct image segmentation. Approximately 2000 images and 250 images were extracted from different experimental videos and used as the training set and validation set, respectively, to train the neural network. Figure 4c shows the loss function curve of the neural network, which indicates the performance of the image process. In this subfigure, one epoch represents that all the training sets are used once. The loss values of the training set and validation set decrease with similar trends, and both loss values converge well after training for 100 epochs. The training and validation curves almost coincide with each other, indicating that the neural network does not suffer from overfitting. Moreover, the training accuracy and validation accuracy are 0.9857 and 0.9790, respectively. Compared with that of traditional manual processing and other image processing methods, the test accuracy is 0.9820. These results indicate that our neural network method is much better and more optimized. A simple procedure of oocyte image segmentation is shown in Fig. 4d. The original input image sequence presents representative photomicrographs of the oocyte volume response. The segmented images can be obtained by inputting the original images into the trained neural network. The white portion presents the shape and area of the oocyte. A contour image sequence is drawn in the corresponding input image based on the black-and-white boundary of the segmented image to visualize the coincidence degree between the original images and the segmented images. The results indicate that the input images and the segmented images have very high coincidence, which proves the accuracy of the neural network method. In general, neural network algorithms significantly improve the accuracy and efficiency of data processing.

### Oocyte volume responses to CPA, determination of mouse oocyte membrane permeability and its CPA concentration dependence

**Fig. 5:**
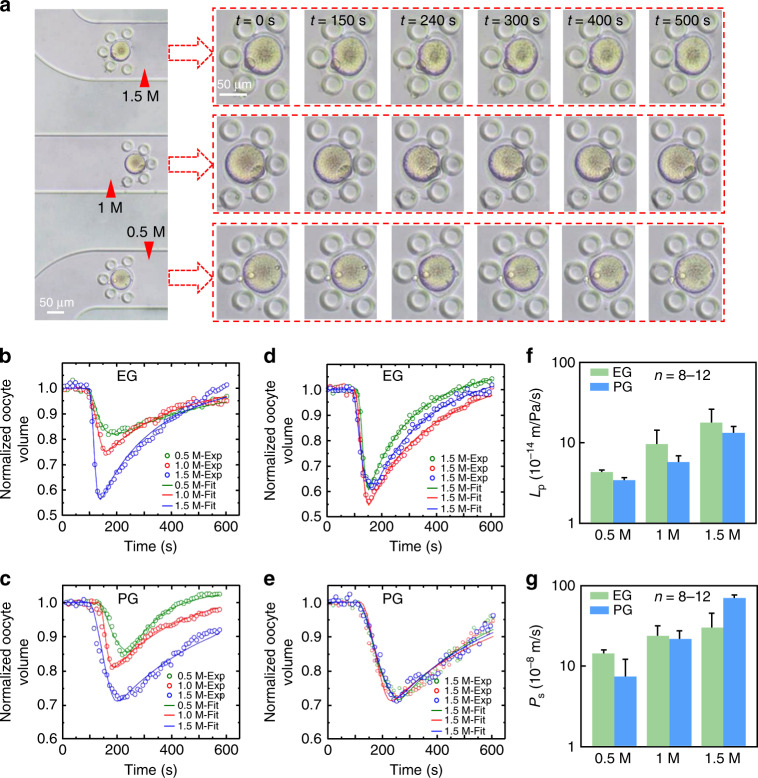
Oocyte volume responses upon CPA replacement and determination of oocyte membrane permeability. **a** Representative photomicrographs of oocyte volume responses to different concentrations of CPA (0.5, 1, and 1.5 M for the three channels). **b**, **c** Normalized oocyte volume changes of representative oocytes exposed to 0.5, 1, 1.5 M EG and PG, respectively. **d**, **e** Normalized oocyte volume changes of representative oocytes exposed to 1.5 M EG and 1.5 M PG, respectively. **f**, **g** Values of *L*_p_ and *P*_s_ of different concentrations of CPA (EG and PG), respectively.

To study the effects of different concentrations and kinds of CPA on oocyte membrane permeability, experiments were performed at room temperature (23 °C) using three different concentrations of ethylene glycol (EG) and 1,2-propanediol (PG), separately. Representative micrographs of the oocyte volume response to different CPA concentrations are shown in Fig. 5a. There is one oocyte captured in each channel. It can be seen that the volume of oocytes undergoes a process of shrinking and then expanding. Owing to the initial difference in the chemical potential of intra- and extracellular water, oocytes were first dehydrated and then gradually rehydrated because of the effect of the cotransport of water and CPA. Nevertheless, the oocyte volume response with time is slightly different because the concentrations in the three channels are different from each other. Moreover, oocytes in three channels were simultaneously added to the same concentration of CPA solution, and the corresponding representative micrographs are shown in Fig. S5. In this study, to avoid oocyte deformation by external mechanical stress, slow mixing was adopted when perfusing CPA solution. The zona pellucida surrounding the oocyte provides a good supporting effect to prevent the closed oocyte membrane from being deformed by external mechanical stress. In addition, any oocytes with irregular or aspherical volume changes were not selected during data processing to ensure measurement accuracy.

Figure 5b, c shows the normalized volume changes of representative oocytes exposed to EG and PG at three different concentrations and the corresponding curve fitting results (lines) with a two-parameter (2-*p*) transport model. In addition, the normalized volume changes of representative oocytes exposed to the same CPA concentrations (1.5 M EG and 1.5 M PG) are shown in Fig. 5d, e. The normalized volume can eliminate the influence of the volume difference of oocytes. As observed from the results, for a given CPA, the volume changes caused by different concentrations are not the same, and for the same concentration, the volume changes caused by different CPAs are also different. Therefore, prior to the addition and removal of CPA, it is necessary to consider not only the concentration added at each step but also the type of CPA used. For EG and PG, the change in oocyte volume is relatively low at the concentration of 0.5 mol/L, causing an insignificant effect on oocytes. However, at concentrations of 1 and 1.5 mol/L, the change in oocyte volume is intense, which causes a negative impact. Moreover, the change in the oocyte volume due to EG is more intense than that with PG. The above results show that for the addition of EG to oocytes, a lower concentration step should be adopted to avoid excessive damage. By fitting the experimental volume change data, the permeability coefficient *L*_p_ of the oocyte membrane to water and the permeability coefficient *P*_s_ to the CPA can be obtained, and the specific values are shown in Fig. 5f, g. It can be seen that at the same temperature, the permeability of the oocyte membrane to different kinds of CPA is different. In addition, the determined *L*_p_ and *P*_s_ of mouse oocytes with different concentrations of EG and PG are listed in Table S2. It can be seen that the permeability values of oocytes increase with increasing concentration. The permeability (*L*_p_ and *P*_s_) of the mouse oocyte membrane to EG and PG reported in the literature using the standard technique is also listed in Table S3 for comparison with the values of this study.

### Fertilization and developmental potential analysis after the experiment

**Fig. 6:**
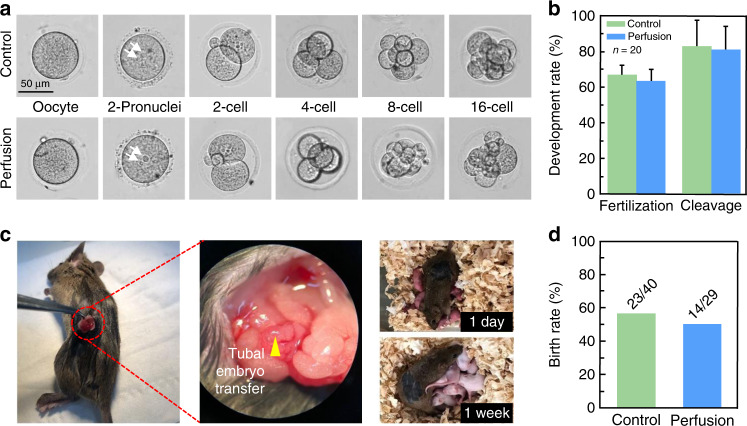
Fertilization, development and reproduction of oocytes for both fresh (control) and perfused groups. **a** Morphology of the mature oocytes and embryo at the 2PN stage, 2-cell stage, 4-cell stage, 8-cell stage, and 16-cell stage. **b** Comparison of embryo development capacity between the control and perfused groups. **c** Photographs of embryo transfer surgery and born mice. **d** Born mice and comparison of natality between the control and perfused groups.

To investigate whether the microfluidic chip and the approach that we used impair the physiological function of the oocytes, in vitro fertilization (IVF) was performed to inseminate the oocytes postperfusion to assess the developmental potential. The fertilized oocytes were cultured for both the control group and the perfusion group. As presented in Fig. 6a, phase contrast micrographs show the morphologies of the mature oocytes and embryo at the 2PN stage, 2-cell stage, 4-cell stage, 8-cell stage, and 16-cell stage. There was no visible difference in terms of the morphology and development capacity of the perfused oocytes compared with the control group. In addition, there was no statistically significant difference in the fertilization and cleavage rates between the two groups (Fig. 6b). These results are attributed to the slow and steady flow rate of the perfusion fluids, which can alleviate osmotic stress. This approach has a very important protective effect on oocytes because oocytes are much more sensitive to osmotic shock. The developmental potential verification indicates that our microfluidic chip and method are feasible and safe for oocytes.

To further confirm that the procedure used in this study is safe and valid, an in vitro embryo transfer study was also conducted. The 2-cell embryos obtained from perfused oocytes and fresh oocytes were transferred into the oviducts of pseudopregnant females. Figure 6c shows a photo of embryo transfer surgery. Approximately 3 weeks after the embryo transfer, offspring were produced. Images of the baby mice 1 day and 1 week after birth are also shown in Fig. 6c; the mice grew well, and there were no abnormalities. The birth rates of the control group and perfusion group (Fig. 6d) were 57.5% vs. 48.3%, respectively, and no obvious difference was observed. The rates of fertilization, cleavage and reproduction of the control group and the perfusion group are listed in Table S4. Offspring production further demonstrates the practicability and effectiveness of the microfluidic chip and research method.

## Discussion

The above measured membrane permeability parameters can further be utilized to optimize oocyte cryopreservation, reproductive pharmacology, etc. For example, during cryopreservation of oocytes, to avoid osmotic damage, it is common to use a stepwise CPA addition/removal protocol. However, this protocol prolongs the exposure time of the oocytes to CPA, which will cause severe cytotoxic damage. Using the determined *L*_p_ and *P*_s_ values, we can predict cell migration during CPA addition/removal and develop an optimal CPA protocol to minimize osmotic damage and cytotoxicity to oocytes. In addition, this approach can help to minimize solute damage and intracellular ice formation by optimizing the cooling rates^45^. Understandably, measurement of membrane permeability parameters plays a vital role in achieving successful oocyte cryopreservation. Furthermore, such measurements can help to select the optimal CPA for a particular cell type, helping to optimize the addition and removal of CPA. We are confident that the current approach also has great prospects in the cryopreservation of other cells.

In previous studies, the permeability of the cell membrane to water and CPA was considered constant and independent of CPA concentration^44^. However, there is evidence that the water permeability decreases with increasing CPA concentration^46,47^. The CPA concentration dependence on permeability has received less attention in the literature, and these studies have yielded conflicting conclusions. Moreover, there has been no study on the concentration dependence of mouse oocyte membrane permeability. According to the results of this study, the permeability of the oocyte membrane to water and CPA both increases as the concentration increases, which is different from other cells. Presumably, this result may be because oocytes contain more intracellular water because of their larger size. At a certain concentration of CPA, the permeability of the oocyte membrane is positively correlated with the concentration of CPA. Therefore, the permeability of the oocyte produces a different result from other cells. Nonetheless, further research is required for verification.

In conclusion, accurate characterization of the permeability of the oocyte membrane is of great significance to the investigation of oocyte cryopreservation, assisted reproduction, and reproductive pharmacology. Here, a specific microfluidic chip was developed to synchronously and nondestructively measure the permeability of multiple mouse oocytes. The chip integrates three independent microchannels, which automatically capture oocytes and simultaneously measure three oocytes exposed to different or identical osmotic shifts. This chip was successfully used to investigate the effect of CPA concentration on oocyte membrane permeability, and it greatly improved the measurement efficiency. Meanwhile, the adopted neural network algorithm significantly enhances the data processing efficiency. The results indicate that the permeability of the mouse oocyte membrane is positively correlated with CPA concentration over a range of CPA concentrations. In addition, this chip is easy to manufacture and is safe for oocyte developmental capacity. This novel microfluidic chip approach has important application prospects in fertility preservation, reproductive medicine, and reproductive pharmacology.

## Materials and methods

### Reagents and ethical statement

All the required reagents used were purchased from Sigma-Aldrich (St. Louis, MO, USA) unless otherwise stated. Pregnant mare serum gonadotropin (PMSG) and human chorionic gonadotropin (hCG) were purchased from Ningbo Second Hormone Factory (Zhejiang, China).

The study was approved by the Institutional Animal Care and Use Committee of the University of Science and Technology of China (USTCACUC1801045).

### Source and preparation of oocytes

Experimental mice (KM 202) were purchased from Beijing Vital River Laboratory Animal Technology Co., Ltd. (Beijing, China). Initially, female mice (6–8 weeks of age) were superovulated with 10 IU PMSG, and 10 IU hCG was administered by intraperitoneal injection 48 h later; 14–16 h after hCG administration, mouse oviducts were collected to obtain oocytes. Cumulus–oocyte complexes (COCs) were released from the ampullary region of each oviduct using a 28-gauge needle. Then, the COCs were incubated in DMEM containing 80 IU/mL hyaluronidase at 37 °C for up to 3 min and further washed 3–4 times with DMEM to obtain clean oocytes. Oocytes were then transferred and cultured in gamete buffer medium (K-SIGB 50, Cook Medical, USA) at 37 °C and 5% CO_2_ until further experimental use.

### Design and fabrication of the microfluidic chip

The microfluidic chip mainly contains a concentration gradient generation region and oocyte capture region. This chip is fabricated using a photoresist mold protocol. Briefly, to create the master mold, a negative photoresistor (SU8 3035, Microchem, USA) is used to create the structure of the microchannel via a lithography procedure on a silicon wafer. It should be pointed out that a two-step spin-coating and three-step exposure method was utilized. After the mold was completed, polydimethylsiloxane (PDMS; Sylgard 184, Dow Corning, Michigan, USA), prepolymer, and curing agent (10:1) were poured onto the aforementioned mold and cured at 78 °C for ~3 h. Then, the chip was removed from the mold, and holes were drilled. Finally, the chip was bonded to a glass slide for experimental use.

### Modeling and simulation of the microfluidic chip channels

To verify the reliability and practicability of the chip, a FEM using COMSOL Multiphysics software was introduced to simulate the microfluidic channel. The volume controller of the model contains the continuous fluid in the two parallel flow tubes, and their inlet and outlet align with the microfluidic channel. FEM requires discretization of the domain, so a user-controlled mesh (free triangular) was utilized to solve this model.

When two or more fluids flow together in the microchannel, the fluid will flow forward side by side, and the mass transfer will be carried out by diffusion under the pressure or concentration difference. Therefore, the concentration change model in the microfluidic channel can be regarded as a laminar flow with the transport of diluted species model. In the simulation, the single-phase laminar flow can be calculated using the Navier–Stokes equation (N–S equation), and the equation for the incompressible flow is as follows^48^:

1 $$\rho \frac{{\partial u}}{{\partial {\mathrm{t}}}} + \rho (u \cdot \nabla )u = \nabla \cdot \left[ { - p{\rm{I}} + \eta \left(\nabla u + \left( {\nabla u} \right)^T\right)} \right] + F$$

2 $$\rho \nabla \cdot u = 0$$

Equation (1) represents the conservation of momentum for incompressible laminar flow. The solution of the equation is always solved together with Eq. (2), which is known as the mass conservation equation. In the equation, *ρ* represents the density of the fluid, *η* represents the dynamic viscosity, *u* denotes the fluid flow rate, *p*I represents the X, Y, and Z axis pressures, and *F* represents the external force acting on the fluid.

The channel wall boundary condition of the microchannel is set to no slip:

3 $$u = 0$$

The boundary conditions at the inlet and outlet of the microchannel are:

4 $$u = {\mathrm{ - }}u_0{\boldsymbol{n}}$$

where *u*_0_ represents the flow rate, and *n* represents the normal unit vector. Two important parameters to consider before calculations are the viscosity and diffusion coefficient of the solution. The CPA solutions that we use are 1.5 mol/L EG and PG, and via the Stokes–Einstein equation (SE equation), we can find the diffusion coefficient *D*_s_:

5 $$D_{\mathrm{s}} = \frac{{k_{\mathrm{B}}T}}{{6\pi \eta r}}$$

where *η* is the viscosity, *k*_B_ is the Boltzmann constant, *r* is the van der Waals radius of the solute molecule, and *T* is the temperature.

The transport of diluted species means that the physical properties such as the density and viscosity of the solution do not change with the uneven distribution of the concentration. At both inlets, the boundary conditions of the concentrations were set to 0.5 and 1.5 mol/L. The boundary condition of the outlet is set to be negligible perpendicular to the boundary, i.e.,

6 $$- n \times D_i\nabla c_i = 0$$

### Oocyte perfusion experiment and data processing

DMEM solution was used as an isotonic solution, and the CPA solutions of EG and PG were used with different concentrations of 0.5 and 1.5 mol/L, separately. All solutions were filtered beforehand to remove impurities. Before the experiment, the most important step is to drain out the bubbles from the channel because oocytes can be constrained during the perfusion process, causing the oocytes to be deformed and damaged. In addition, the bubbles can affect the observation of certain parameters during the experiment, such as volume change, which cannot be observed. To drain out the bubbles, the flow rate of the syringe pump was set as 30 μL/min to make the isotonic solution fill the channel. After all bubbles were eliminated, the oocytes in the syringe were loaded with a hose, and then, oocytes were injected into microchannels through the three cell inlets. When the oocytes were captured by the microcolumn array in the three channels, the CPA solution perfusion experiment was performed, and the flow rate was 10 μL/min. There are also three syringe pumps used to collect the waste solution. Here, three different (Inlet1: 1.5 M, Inlet2: 0.5 M) or identical (Inlet1: 1.5 M, Inlet2: 1.5 M) osmotic shifts may be produced and applied to the oocytes captured in the channels by introducing different combination of CPA solutions from the two inlets. The microfluidic chip was placed under an inverted microscope with an industrial camera to view and record the whole oocyte capture region. The experimental temperature was room temperature (23 °C).

To precisely and quickly process the experimental data, a neural network method was adopted to analyze these images^39,49,50^. Prior to using the neural network, datasets for training should be made. In brief, each recorded video was cut into three subvideos with 256 × 256 pixels, each containing one oocyte. Then, the subvideo was converted into a series of images, and partial images were selected for further processing. These selected images manually circled the shape of the oocyte membrane. To accurately measure the permeability of the oocyte membrane, the oocyte membrane manually circled only contains cytoplasm, without zona pellucida. Then, the pixel values of the oocyte and other positions in the image were set to 255 and 0, respectively, to make a gray image as the output label of the network. The theory and procedure of this neural network approach are described in detail elsewhere^39,49^. After processing by the neural network, the obtained data were calculated to obtain the oocyte volume value. Finally, the normalized volume data were substituted into the 2-*p* equation^51^, and the mouse oocyte membrane permeability coefficient was obtained by fitting^51^.

### In vitro fertilization, embryo culture, and embryo transfer

For IVF, male KM mice older than 8 weeks were euthanized by cervical dislocation, and sperm were collected by dissecting the epididymis. The sperm were put into 1 mL of equilibrated sperm washing solution, the epididymis tissue was removed, and the sperm were incubated at 37 °C in 5% CO_2_ air for 20–25 min to make the sperm free swimming. Then, 10 µL of the abovementioned preliminarily incubated and well-dispersed active sperm suspension was added to 50 µL of equilibrated washing solution and incubated for 2 h at 37 °C, 5% CO_2_ and saturated humidity. Five oocytes were inseminated with 2 × 10^4^ sperm for ~5 h in a 100-µL droplet of fertilization medium (K-SIFM 50, Cook Medical, USA). Subsequently, fertilized oocytes were selected for culture in a drop of 50 µL of cleavage medium (K-SICM 50, Cook Medical, USA) at 37 °C in 5% CO_2_. In the following days, the development of the fertilized oocytes, that is, the formation of 2-cell, 4-cell, 8-cell, and 16-cell embryos, was monitored and recorded by optical microscopy.

Prior to embryo transfer, female mice (B6/CBA) were copulated with vasectomized male mice as pseudopregnant mice. Tubal embryo transfer (TET) was adopted to implant 2-cell embryos. During surgery, the pseudopregnant mice were anesthetized, the hair on the back of the surgical area was removed, and a 10 mm longitudinal incision was cut. The ovaries, fallopian tubes and part of the uterus were removed by routine operation. After cutting the fallopian tube, the microtubule-containing embryo was inserted into the incision, and the embryo was injected. Subsequently, the ovary was gently pushed into the body, and the wound was sutured. The mice were kept warm after surgery until they woke up, waiting for the birth of the baby mice.

## Supplementary information

Supplemental Material
