# Supplementary material for: A microfluidic approach for synchronous and nondestructive study of the permeability of multiple oocytes
Source: Microsyst Nanoeng. 2020 Jul 27;6:55. doi: 10.1038/s41378-020-0160-4 (PMC8433209; doi:10.1038/s41378-020-0160-4)
Supplement: Supplementary file 1 — Supplemental Material [file 41378_2020_160_MOESM1_ESM.docx]

**Supporting Information**

**A Microfluidic Approach for Synchronous and Nondestructive Study of Permeability of Multiple Oocytes**

Zhongrong Chen^1^, Kashan Memon^1^, Yunxia Cao^2,3,*^, Gang Zhao^1,3,*^

^1^Department of Electronic Science and Technology, University of Science and Technology of China, Hefei, Anhui 230027, China

^2^Reproductive Medicine Center, Department of Obstetrics and Gynecology, The First Aﬃliated Hospital of Anhui Medical University, Hefei 230022, Anhui, China

^3^Anhui Province Key Laboratory of Reproductive Health and Genetics, Anhui Provincial Engineering Technology Research Center for Biopreservation and Artiﬁcial Organs, Anhui Medical University, Hefei 230022, Anhui, China

* Correspondence should be addressed to: Gang Zhao, Ph.D.

Department of Electronic Science & Technology

University of Science and Technology of China

Road Jinzhai 96, Hefei 230027, Anhui, P. R. China

Tel: +86-18256929838

E-mail: [zhaog@ustc.edu.cn](mailto:zhaog@ustc.edu.cn); [caoyunxia6@126.com](mailto:caoyunxia6@126.com)

**Supplementary Figures**

Figure S1. A schematic of the entire experimental system.

Figure S2. Modeling of microfluidic serpentine channel.

Figure S3. Comparative simulation study of different designs of oocytes capture region.

Figure S4. Characterization of solution concentration change in microfluidic channels.

Figure S5. Oocytes volume response upon CPA replacement.

**Supplementary Tables**

Table S1. Simulated prediction of concentration profile on specific points.

Table S2. Determined *L*_p_ and *P*_s_ of mouse oocyte with different concentration EG and PG.

Table S3. Mouse oocyte membrane permeability coefficients under different condition.

Table S4. Rate of fertilization, cleavage and reproduction of control group and perfusion group.

**
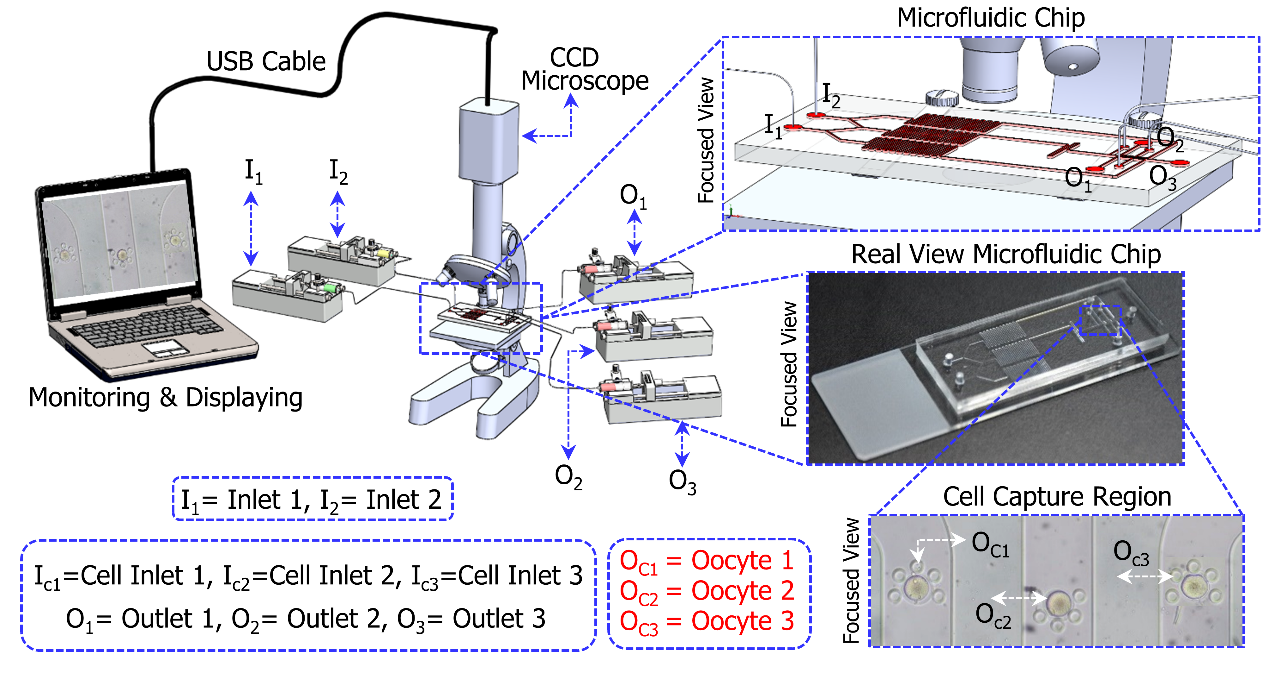
**

**Figure S1.** A schematic of the entire experimental system.


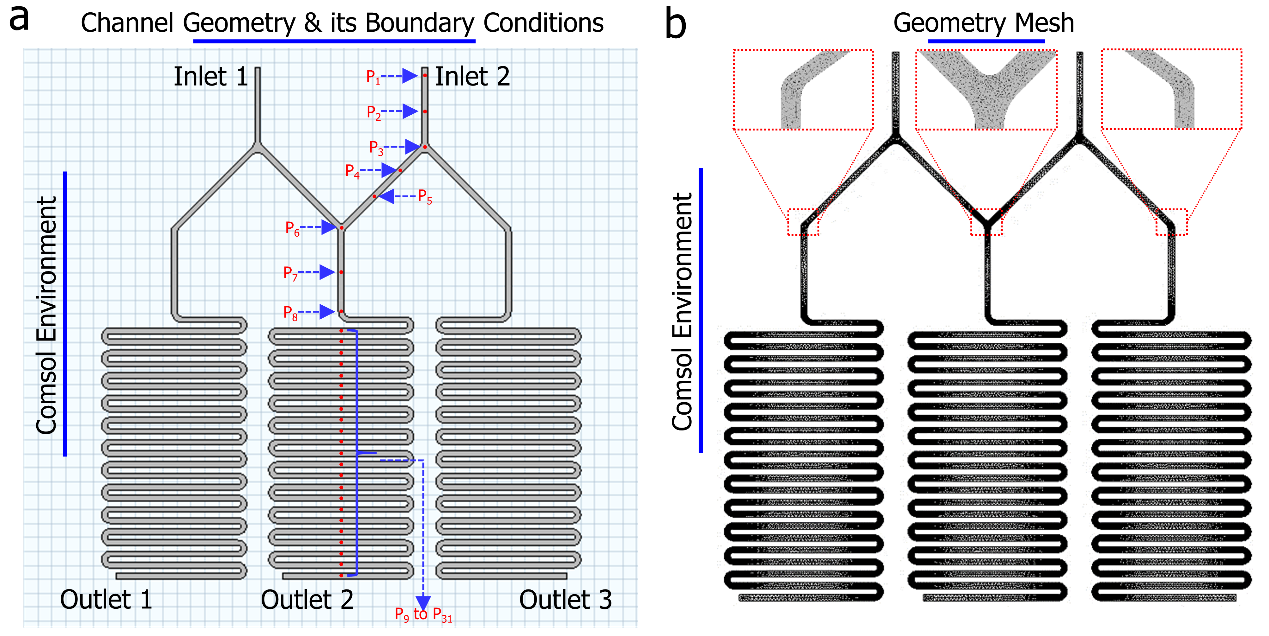


**Figure S2.** Modeling of microfluidic serpentine channel. (a) Physical model of microfluidic serpentine channel geometry. (b) Mesh geometry of simulated domain.


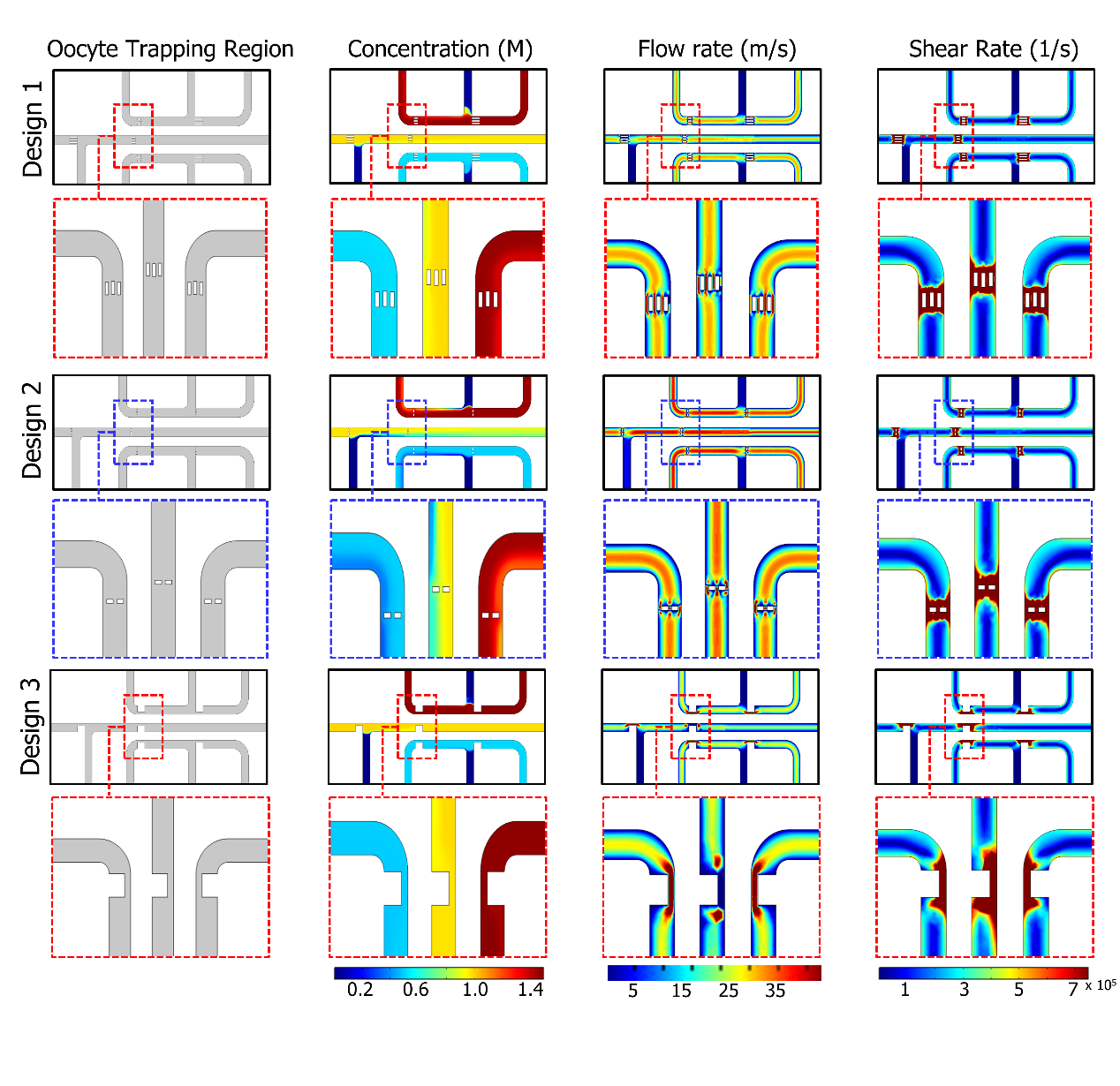


**Figure S3.** Comparative simulation study of three other designs of oocytes capture region. Including concentration profile, flow rate and shear rate.

**
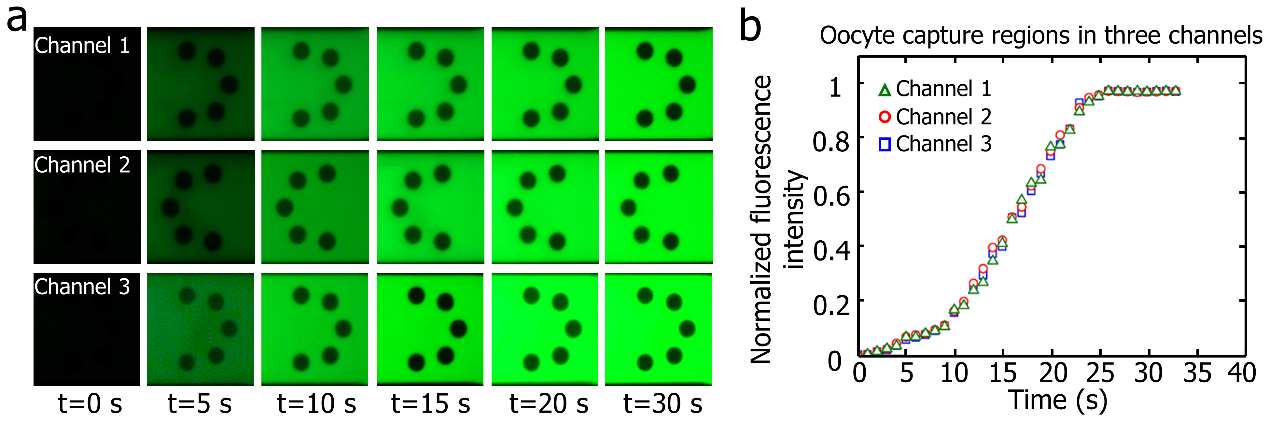
**

**Figure S4.** Characterization of solution concentration change in microfluidic channels.

(a) The fluorescence map corresponding to three oocyte capture locations with the same concentration. (b) Change in fluorescence intensity at three oocyte capture locations.


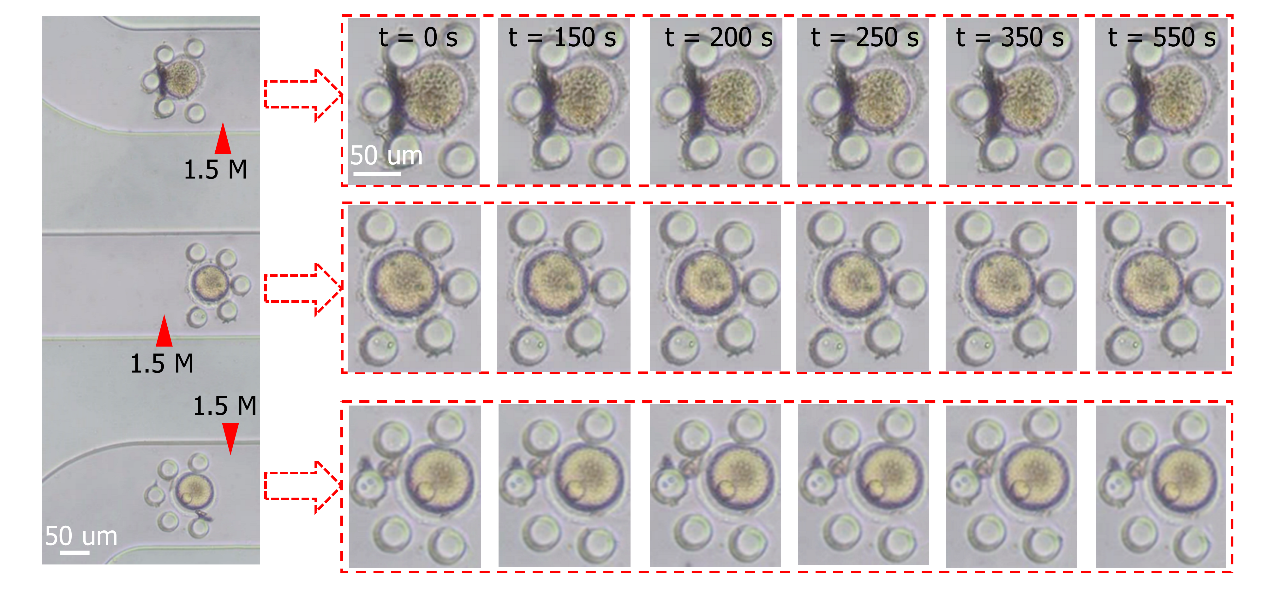


**Figure S5.** Oocytes volume response upon CPA replacement. Representative photomicrographs of oocytes volume responses to the same concentration CPA (1.5 M for 3 channels)

**Table S1.** Simulated prediction of concentration profile on specific points

| *No. of Points* | *Coordinates (x(mm), y(mm))* | *Concentration（mol/L）* |
| --- | --- | --- |
| P_1_ | (26.9, 20.6) | 1.500 |
| P_2_ | (26.9, 19.2) | 1.500 |
| P_3_  P_4_  P_5_  P_6_  P_7_  P_8_  P_9_  P_10_  P_11_  P_12_  P_13_  P_14_  P_15_  P_16_  P_17_  P_18_  P_19_  P_20_  P_21_  P_22_  P_23_  P_24_  P_25_  P_26_  P_27_  P_28_  P_29_  P_30_  P_31_ | (26.9, 18)  (26.9, 17.4)  (25.4, 16.6)  (23.9, 15.1)  (23.9, 13.5)  (23.9, 12)  (23.9, 11.4)  (23.9, 11)  (23.9, 10.6)  (23.9, 10.2)  (23.9, 9.8)  (23.9, 9.4)  (23.9, 9)  (23.9, 8.6)  (23.9, 8.2)  (23.9, 7.8)  (23.9, 7.4)  (23.9, 7)  (23.9, 6.6)  (23.9, 6.2)  (23.9, 5.8)  (23.9, 5.4)  (23.9, 5)  (23.9, 4.6)  (23.9, 4.2)  (23.9, 3.8)  (23.9, 3.4)  (23.9, 3)  (23.9, 2.6) | 1.500  1.471  1.440  1.409  1.378  1.347  1.316  1.285  1.283  1.254  1.223  1.193  1.095  1.048  1.001  1.000  1.000  1.000  1.000  1.048  1.095  1.083  1.000  1.000  1.000  1.000  1.000  1.000  1.000 |

**Table S2.** Determined *L*_p_ and *P*_s_ of mouse oocytes with different concentration EG and PG

| CPA | Concentration（mol/L） | *L_p_*（10^-14^ m/Pa/s） | *P_s_*（10^-8^ m/s） |
| --- | --- | --- | --- |
|  | 0.5 | 4.16 ± 0.27 | 14.69 ± 1.49 |
| EG | 1 | 9.25 ± 4.61 | 24.48 ± 7.34 |
|  | 1.5 | 17.04 ± 8.39 | 30.06 ± 15.06 |
|  | 0.5 | 3.30 ± 0.23 | 7.50 ± 4.90 |
| PG | 1 | 5.61 ± 1.08 | 21.74 ± 6.04 |
|  | 1.5 | 12.75 ± 2.48 | 71.24 ± 6.39 |

**Table S3.** Mouse oocyte membrane permeability coefficients under different condition.

| CPA (mol/L) | *L_p_*（μm/atm/min） | *P_s_*（μm/s） | T (°C) | Refs. |
| --- | --- | --- | --- | --- |
| EG (0.5) | 0.25 ± 0.02 | 0.15 ± 0.01 | 23 °C | Present |
| EG (1) | 0.56 ± 0.28 | 0.24 ± 0.07 |  |  |
| EG (1.5) | 1.02 ± 0.50 | 0.30 ± 0.15 |  |  |
| PG (0.5) | 0.20 ± 0.01 | 0.08 ± 0.05 |  |  |
| PG (1) | 0.34 ± 0.06 | 0.21 ± 0.06 |  |  |
| PG (1.5) | 0.77 ± 0.15 | 0.71 ± 0.06 |  |  |
| EG (1.5) | 0.43 ± 0.11 | 0.32 ± 0.15 | 25 °C | 1 |
| PG (1.5) | 0.48 ± 0.18 | 0.52 ± 0.16 |  |  |
| EG (1.5) | 0.73 ± 0.14 | 0.52 ± 0.16 | 37 °C |  |
| PG (1.5) | 0.66 ± 0.12 | 1.01 ± 0.17 |  |  |
| EG (1.5) | 0.51 ± 0.08 | 0.09 ± 0.02 | 20 °C | 2 |
| PG (1.5) | 0.36 ± 0.13 | 0.24 ± 0.13 |  |  |
| EG (1.5) | 0.91 ± 0.16 | 0.24 ± 0.05 | 30 °C |  |
| PG (1.5) | 0.53 ± 0.05 | 0.43 ± 0.12 |  |  |
| EG (1.6) | 0.42 ± 0.06 | 0.10 ± 0.01 | 25 °C | 3 |
| PG (1.3) | 0.48 ± 0.13 | 0.28 ± 0.10 |  |  |

**Table S4.** Rate of fertilization, cleavage and reproduction of control group and the perfusion group.

|  | Oocyte  number | Fertilization rate (%) | | Cleavage rate (%) | | Birth rate  (%) |
| --- | --- | --- | --- | --- | --- | --- |
|  |  | Groups | Average ± SD | Groups | Average ± SD |  |
| Control group | 39 | 72.73 (8/11) | 66.95 ± 5.31 | 87.50 (7/8) | 82.71 ± 14.47 | 57.5  (23/40) |
|  |  | 71.43 (5/7) |  | 60.00 (3/5) |  |  |
|  |  | 60.00 (6/10) |  | 83.33 (5/6) |  |  |
|  |  | 63.64 (7/11) |  | 100.00 (7/7) |  |  |
| Perfused group | 52 | 62.50 (5/8) | 63.48 ± 6.66 | 100.00 (5/5) | 80.63 ± 13.27 | 48.3  (14/29) |
|  |  | 53.33 (8/15) |  | 62.50 (5/8) |  |  |
|  |  | 66.67 (10/15) |  | 80.00 (8/10) |  |  |
|  |  | 71.43 (10/14) |  | 80.00 (8/10) |  |  |

**References**

1. Lei, Z. *et al.* A microfluidic platform with cell-scale precise temperature control for simultaneous investigation of the osmotic responses of multiple oocytes. *Lab Chip* **19**, 1929-1940 (2019).
2. Paynter, S. J. A rational approach to oocyte cryopreservation. *Reprod Biomed Online* **10**, 578-586 (2005)
3. Edashige, K. *et al*. The Role of Aquaporin 3 in the Movement of Water and Cryoprotectants in Mouse Morulae, *Biol Reprod*, **77**, 365-375(2007).
